# A Warm Discussion

**Published:** 1897-02

**Authors:** 


A WARM DISCUSSION.—It will be seen by reference to our original articles—(Abortion; Discussion, etc.), that Dr. Q. Cincinnatus Smith, of Austin, after outlining a very rational treatment for abortion, takes his colleague, Dr. W. J. Mathews, to task—seriously and literally;—quoting him as saying that "any physician who would not mop out the uterine cavity with carbolic acid, full strength, after an abortion, should be indicted for manslaughter in case of the death of the woman." He jumps on him, figuratively, with both feet, and metaphorically "knocks the stuffin' out of him." He is wrathy! His denunciation is something terrific.

Now— we do not hardly believe that Dr. Mathews (who is a kind-hearted man), really meant what he said, or is said to have said. He must have had some mental reservation—(he expressed none), and was, most likely, articulating through the interstices of his Derby—; or, he may have meant it in a Pickwickian sense. We hardly believe that Dr. Mathews could be so cold-blooded and so hard-hearted as to see good old Dr. Pray—whom Dr. Smith instances, figuratively, as having dared to differ with Brother Mathews on treatment,—condemned to the pen, for manslaughter, just because he did not treat a case of abortion by the rule of thumb formulated by Dr. Mathews; hardly. He could not go so far. In the fiery zeal of his youthful patriotism, (this was early in the war), Bill exclaimed "Victory or death." (That was just before the battle—mother—,) and turning to a companion-in-arms, said, "don't you say so Jo?" "Well," said Jo, "*hardly;* I *might* go—"victory or—*wounded*," but—death? Not any for Josephus!" We venture, now that the cooling influences of time and reflection have succeeded the heat of forensic disquisition—and his mind is a little clearer perhaps, if Dr. M. were asked, "what should be done with old Dr. Pray?—(hypothetical case; assuming, as the lawyers say, all the counts in the indictment to have been proven); would you hang him? "Oh, no, no. I'd give him some advice, and say, "go slow."

It is strange how great minds will differ. Now—this great mind—yours truly, ye writer, ye senior—if called on for an opinion on the subject of the use of pure carbolic acid applied to the endometrium—would have been rather inclined to say—as Mr. Bumble did of the law—that any man who would do it "is a hass"—with a capital H. Yet, had we expressed any such opinion, we know we should have been very severely censured.

If Dr. M. had gone a little farther back,—and said—in speaking of abortion—any man who would procure an abortion for other than strictly legitimate reasons—ought to be *hanged*, in this sentiment I am sure Dr. Smith and every other honorable doctor would have heartily agreed with him, and there would have been furnished abundant additional matter for a display of the good doctor's choice invective. But—the cause of the abortion was not the subject of discussion; that's another story.

